# Sport-Related Injuries in Portuguese CrossFit^®^ Practitioners and Their Characteristics

**DOI:** 10.3390/muscles4010002

**Published:** 2025-01-10

**Authors:** Ricardo Maia Ferreira, Luís Gonçalves Fernandes, Beatriz Minghelli, Yuri Feito, António Rodrigues Sampaio, Nuno Pimenta

**Affiliations:** 1 Social Sciences, Education and Sport Department, Polytechnic Institute of Maia, N2i, 4475-690 Maia, Portugal; lfernandes@ipmaia.pt (L.G.F.); arsampaio@umaia.pt (A.R.S.); d011557@ipmaia.pt (N.P.); 2 Physioterapy Department, Coimbra Health School, Polytechnic Institute of Coimbra, 3046-854 Coimbra, Portugal; 3 Sport Physical Activity and Health Research & Innovation Center (SPRINT), 4960-320 Melgaço, Portugal; 4 Jean Piaget Algarve Health School, Piaget Institute, 8300-025 Silves, Portugal; beatriz.minghelli@ipiaget.pt; 5 Research Unit in Human Movement (KinesioLab), 1950-157 Lisboa, Portugal; 6 Nea Onnim Consultancy, FL, USA; yfeito@gmail.com; 7 Research Center in Sports Sciences, Health Sciences and Human Development (CIDESD), Department of Physical Education and Sports Sciences, University of Maia, 4475-690 Maia, Portugal

**Keywords:** CrossFit^®^ training, injuries, epidemiology

## Abstract

Background/Objectives: CrossFit^®^ is one of the most popular yet controversial training regimens. Some groups extol the positive effects of its practice, while others argue that it is unsafe and that there is limited information. The aim of this study was to investigate, through a self-reported questionnaire, the epidemiology of Portuguese CrossFit^®^ training practitioners. Methods: Statistical analyses, including Mann–Whitney U, chi-square, Spearman’s rho correlations, and logistic regressions, were conducted. Results: A total of 288 practitioners completed the questionnaire, with 39.9% reporting injuries. These injuries occurred mainly during training, particularly when performing gymnastics exercises. Tendon (40.9%) and shoulder (46.1%) were the most common injuries. Key risk factors for injury included male gender (OR = 2.7), years of practice (4–6 years: OR = 7.22), heavier body weight (OR = 4.76), and higher weekly training volume (4–6 times per week). Conclusions: Approximately 40% of CrossFit^®^ practitioners are expected to experience injuries, particularly in the shoulder and tendons. Injury risk is influenced by factors such as practice years, weekly training, sex, and body weight. These findings may help guide practitioners, coaches, and health professionals in better understanding the risk factors, mitigating injuries, and developing effective injury prevention strategies.

## 1. Introduction

Pursuing health and sports activities has become increasingly popular, as physical exercise is recognized as a critical component for maintaining long-term health [1]. Exercise has the potential to enhance or sustain cardiorespiratory, musculoskeletal, and neuromotor health and fitness [2]. People have turned to various activities to gain the benefits that exercise can offer and achieve the best results [3]. The training regimens more often used and prescribed include endurance, resistance, flexibility, and neuromotor exercises, performed at low, moderate, or high intensity [2]. Presently, high-intensity training is among the most prevalent training regimens, as it has demonstrated positive effects on metabolic, mechanical, neuronal, and hormonal systems [4,5]. High-intensity training involves brief, intense activities interspersed with rest periods or low-intensity exercises, encompassing single-joint or multi-joint movements [6,7,8]. Currently, one of the most popular high-intensity multi-joint training regimens is high-intensity functional training [9], more commonly known as CrossFit^®^ training [10].

CrossFit^®^ training is one of the most widespread and rapidly growing training programs globally and was created by Greg Glassman in the year 2000 [11,12]. Initially developed for the military, it quickly spread to the general population, and the first CrossFit^®^ training gym opened in 2000 in California [13]. Since then, the number of members and gyms has grown, with over 15,000 active CrossFit^®^ gyms (or boxes) estimated to exist, and 10,000 daily practitioners across 142 countries [10,11,12,13]. The CrossFit^®^ market is valued at approximately USD 4.8 billion, with an annual growth of 3.4% [14]. In recent years, CrossFit^®^ has continued to develop and become a sport through a worldwide competition—the CrossFit^®^ Games [1,15,16]. In the 2023 Games, 322,000 athletes enrolled in the competition, and the Fittest Man and Woman on Earth each won USD 315,000, with a total payout of USD 2.945 million across all divisions [16]. In Portugal, there are 78 registered CrossFit^®^ gyms [17]. Additionally, there are unregistered gyms/boxes that provide CrossFit^®^ training but do not meet instructor requirements and/or do not pay the affiliate fee to use the brand/name “CrossFit^®^” [18,19]. It is estimated that this value could be as high as 50% [19]. Therefore, the total number of gyms/boxes and practitioners in the country remains unknown.

CrossFit^®^ training is designed to enhance overall physical conditioning and health, based on the principles of exercise variability, high-intensity training, and functional movements [20,21]. Allegedly, CrossFit^®^ training can optimize physical competence in ten fitness domains [13,22]: (1) strength, (2) power, (3) stamina, (4) speed, (5) agility, (6) coordination, (7) balance, (8) accuracy, (9) flexibility, and (10) cardiovascular/respiratory endurance. Usually, training sessions (i.e., “workout of the day” [WOD]) are of short duration (5–20 min) and incorporate diverse exercises, particularly [21,22,23] powerlifting (e.g., squats, deadlifts, presses/push presses, and bench presses); Olympic weightlifting (e.g., clean and jerk, and snatch); gymnastics (e.g., knees-to-elbows, lunges, pull-ups, muscle-ups, toes-to-bar, burpees, gluteus hamstrings, rope climbs, dips, developer sit-ups, handstand pushups, pistols, and push-ups); endurance (e.g., cycling, rowing, running, sprints, and rope jumping,); and others (e.g., thrusters, box jumps, Turkish get-ups, kettlebell swings, and double unders). These exercises are often combined into workout routines performed quickly, repetitively, and with limited or no recovery time, in “best time” or “as many rounds as possible” regimes [23,24,25,26,27].

The WODs are performed in group classes in the boxes (open spaces, with few gym machines [mainly for endurance], equipped with free weightlifting [e.g., weights discs, dumbbells, and kettlebells] and gymnastics [e.g., rings and fixed bar] apparatus) and are usually monitored by certified trainers/instructors [28,29]. Individuals with a variety of skills and abilities can exercise in the same WOD, as a key feature of CrossFit^®^ exercises is scalability—where the loads/movements are adjusted on an individual basis considering the level of skill and conditioning [20,24,25,28]. This group training type is appealing to those practicing it and mobilizes them to exercise, as there is a more peer-supporting atmosphere and an intra/inter-competition factor, which is positive for achieving better results [13,25,28,30,31,32]. This also helps create a sense of community, as members report experiencing significantly more connectedness and friendship compared to other sports [13,25,28,29,30,31,33]. This, associated with strong marketing, has increased its popularity over the years [29].

Despite the expected benefits of practicing it, the safety of CrossFit^®^ training still raises discussion, particularly due to the limited and sometimes contradictory data in the literature regarding its injury incidence, prevalence, nature, mechanisms, and risk factors [11,12,20,22,23,24,25,26,34,35,36,37,38]. In fact, a consensus paper produced by the Consortium for Health and Military Performance and the American College of Sports Medicine associated a potential emergence of high injury risk with extreme conditioning programs, such as CrossFit^®^ training [37]. The consensus highlighted a “disproportionate musculoskeletal injury risk from these demanding programs, particularly for novice participants, resulting in lost duty time, medical treatment, and extensive rehabilitation” [37]. Nonetheless, since the publication of that report, several investigators have argued about the safety of CrossFit^®^ training [10,23,25,34,39]. Furthermore, it is always fundamental to analyze injury patterns, as it allows athletes, coaches, and healthcare professionals to improve their knowledge about injury prevention and health promotion [39]. Additionally, there is limited information about CrossFit^®^ practitioners in Portugal. Only one study has reported injury incidence in the Portuguese population [40]. However, that study is over five years old and included 270 participants from the southern region, limiting its generalizability. Therefore, the purpose of this study was to investigate the epidemiology of CrossFit^®^ training practitioners residing in Portugal. It is hypothesized that the sport-specific movements, skills, and actions in CrossFit^®^ may lead to different injury types among practitioners, with both personal and sport-related factors contributing to the injury’s recurrence patterns.

## 2. Materials and Methods

A quantitative, retrospective, cross-sectional, self-reporting study was conducted following established methodological guidelines [41,42,43,44] using an electronic survey (e-survey). This study adhered to the Ethical Principles of the Helsinki Declaration [45] and received approval from the Polytechnic Institute of Maia Ethics Committee.

Before starting the e-survey, the front page explicitly stated the purpose and context of the study, data protection rights, how the results would be used, criteria for participant selection, and other relevant information. Consent for participation in the study was obtained through an informed consent statement. After providing informed consent and agreeing to participate in the study, CrossFit^®^ training practitioners were granted access to the e-survey.

### 2.1. Participants and Data Collection

Participants were recruited from CrossFit^®^ training gyms and boxes in Portugal. To ensure the correct population sample, potential participants were reached through communication channels of the gyms/boxes. Coordinators/owners/coaches were requested to directly contact their members, explaining the study’s procedures, objectives, and eligibility criteria. Reminders containing the questionnaire link and thank-you notes were sent at 2, 4, and 6 weeks to achieve a sizable sample. Eligibility criteria for participation included proficiency in reading and writing Portuguese, being 18 years or older, of any sex, including both healthy and unhealthy individuals, with any level of CrossFit^®^ experience. Furthermore, participants were required to reside in Portugal (mainland or islands), actively practice CrossFit^®^, sign the informed consent, and complete the questionnaire.

### 2.2. Questionnaire

The questionnaire link was shared with the participants through the Google Forms software (https://www.google.com/forms/about/). Before distributing the e-survey, the questionnaire was developed by reviewing the most recent literature and pre-tested by the authors for completion time, design, question order, attractiveness, syntax, clarity, logic, correct question type, and response format. Additionally, the questionnaire was evaluated by an external expert panel (2 independent and methodologically experienced PhDs with exercise and health backgrounds) and pre-tested in a representative sample for comments and suggestions. The e-survey took 10–15 min to complete and included 29 closed-ended questions, divided into 2 main stages (21 sport and sociodemographic-related items, and 8 health disorders and injury-related items):

1. Sport and sociodemographic-related items. The items covered information regarding age, sex, height, weight, professional physical level, sports background, years of CrossFit^®^ training, CrossFit^®^ training level, CrossFit^®^ training competition participation, CrossFit^®^ training load, training context, and CrossFit^®^ training warm-up and cool-down.
2. Health disorders and injuries-related items. The items covered information regarding health disorders and injuries related to CrossFit^®^ Training, e.g., type, location, frequency, time to return to sport, context, health history, and management. Items in this phase were adapted from a consensus statement on injury registration [46]. It defined injury as any physical damage to a body part, sustained during training or competition, which prevented the participant from training, working, or competing in any way and for any period of time [25,47]. To ensure questionnaires were filled out properly, definitions and examples were given throughout the items, helping to contextualize the readers.

### 2.3. Statistical Analysis

Data from the e-survey were entered into a protected database, and results were determined and displayed in tabular and graphic formats using Microsoft Excel and IBM SPSS 26.0 software. After checking for data normal distribution, chi-square tests (nominal data) and independent two-sample non-parametric Mann–Whitney U (ordinal data) were conducted. Additionally, correlations between the variables were analyzed using Spearman’s rho. The strength of the relationship was assessed based on the following criteria [48]: 0 to 0.20—Negligible; 0.21 to 0.40—Weak; 0.41 to 0.60—Moderate; 0.61 to 0.80—Strong; and 0.81 to 1.00—Very strong. Moreover, logistic regression analyses were conducted following the enter model to examine the associations between CrossFit^®^ training practitioners’ sport/sociodemographic characteristics and injuries. Before examining the associations, certain categories were merged and renamed to ensure stability in logistic regression models. A significance level of 0.05 was used to determine whether a model needed to be reported [49]. For each level of the independent variables, odds ratios (ORs) and their 95% confidence intervals (CIs) were calculated. CIs that included 1.0 were considered not statistically significant. The R-square (R^2^) values were interpreted as follows [50]: R^2^ < 2%—Very weak; 2% ≤ R^2^ < 13%—Weak; 13% ≤ R^2^ < 26%—Moderate; and R^2^ ≥ 26%—Substantial.

## 3. Results

**Figure 1:**
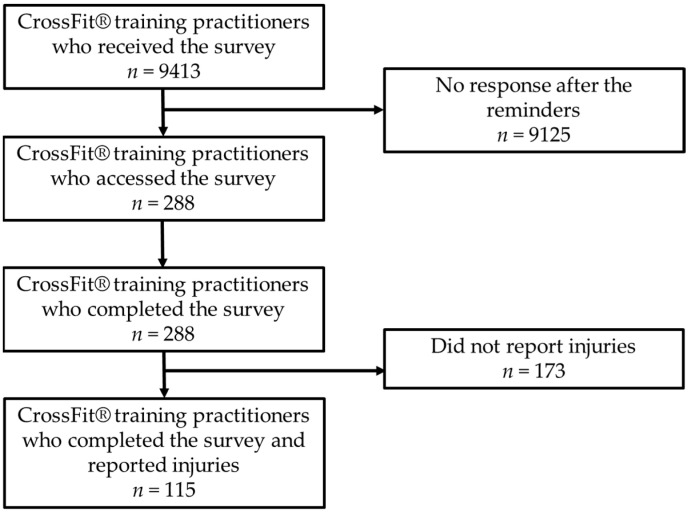
Questionnaire views, participation, and completion.

The e-survey reached 9413 CrossFit^®^ training practitioners, and among them, 288 expressed interest in participating in the study. Figure 1 illustrates the flow diagram of the study.

Among the 288 CrossFit^®^ training practitioners who completed the questionnaire, males were more predominant (148; 51.4%), and the majority fell within the 35–39 age group (69; 24.0%), with body weights ranging from 50 to 74 kg (158; 54.9%) and heights between 1.50 and 1.74 m (180; 62.5%). A sizable portion of respondents had engaged in sports or physical activity before practicing CrossFit^®^ training (219; 76.0%), but currently, 59.0% (n = 170) did not participate in any additional sports or physical activities. The most common job-related physical activity level reported involved sitting and walking without physical effort (113; 39.2%). Most participants had been practicing CrossFit^®^ training for 1–3 years (94; 32.6%) at a non-competitive level (197; 68.4%). The majority trained 4–6 times per week (180; 62.5%), with sessions lasting 30–59 min (61.8%). Typically, warm-ups were performed (232; 80.5%), lasting less than 10 min (178; 48.6%), and comprised mobility exercises, stretching, core exercises, and sport-specific movements/exercises (Table 1). Similarly, cool-downs were performed (189; 65.6%), lasting less than 10 min (163; 56.6%), and consisted of stretching exercises (122; 64.6%). In the sample, most participants were monitored by an instructor (255; 88.5%), but not by a health professional (154; 53.5%). Detailed data on CrossFit^®^ training practitioners’ sport and sociodemographic characteristics can be found in Table 2.

Out of the 288 CrossFit^®^ training practitioners, 115 (39.9%) reported having an injury. The majority reported experiencing one to three injuries (108; 93.9%), during training sessions (96; 83.5%), and while performing gymnastics exercises (35; 30.4%). Shoulder (53; 46.1%) and musculotendinous (79; 68.7%) injuries were the most common localization and types. Specifically, tendon injuries surpassed muscular injuries (47 (40.9%)—total, 29 (54.7%)—shoulder; 32 (27.8%)—total, 13 (24.5%)—shoulder, respectively). The injuries had no clinical history (92; 80%) and were mostly managed with physiotherapy (52; 45.2%), resulting in sport absenteeism of less than 1 week (28; 24.3%)—Table 3, Table 4 and Table 5.

When comparing practitioners of CrossFit^®^ training who reported injuries to those who did not, we identified significant statistical differences in the years practicing CrossFit^®^ training (*p* = 0.006) and weekly CrossFit^®^ training practice (*p* = 0.003) items. Other variables did not exhibit significant statistical differences; refer to Table 1 for additional details.

We also found statistically significant correlations between injuries and various variables. A moderate positive correlation (0.454, *p* ≤ 0.01) emerged between the years practicing CrossFit^®^ training and the number of injuries suffered. Additionally, weak positive correlations (0.248–0.337, *p* ≤ 0.01) were found between the following variables: professional physical activity level vs. the number of injuries suffered; CrossFit^®^ training level vs. injury occurrence situation; annual CrossFit^®^ training competitions vs. injury occurrence situation; injury localization vs. injury CrossFit^®^ training activity. Furthermore, two weak negative correlations between the professional physical activity level vs. injury CrossFit^®^ training activity (−0.249, *p* ≤ 0.01), and years practicing CrossFit^®^ training vs. injury CrossFit^®^ training activity (−0.246, *p* ≤ 0.01) variables were found. Also, a weak positive correlation (0.221, *p* ≤ 0.05) between weight and the number of injuries suffered was identified. Moreover, negligible positive correlations (0.186–0.202, *p* ≤ 0.05) were found between the variables: age vs. number of injuries suffered; professional physical activity level vs. injury occurrence situation; and years practicing CrossFit^®^ training vs. return-to-sport duration. Finally, negligible negative correlations (−0.191–−0.208, *p* ≤ 0.05) were found between the variables: existence and being monitored by a CrossFit^®^ training instructor vs. the number of injuries suffered; the number of injuries suffered vs. injury CrossFit^®^ training activity; injury localization vs. injury occurrence situation; and injury CrossFit^®^ training activity vs. return-to-sport duration.

Non-injury-related items also showed statistically significant correlations. Two very strong positive correlations were identified: one between CrossFit^®^ training level and the number of annual CrossFit^®^ competitions (r = 0.955, *p* ≤ 0.01), and another between CrossFit^®^ training level and the use of cool-down strategies (r = 0.857, *p* ≤ 0.01). Moreover, a strong positive correlation (0.723, *p* ≤ 0.01) was found between the variables sex and height. In addition, moderate positive correlations (0.558–0.605, *p* ≤ 0.01) were found between the variables sex vs. weight and weight vs. height. Also, weak positive statistically significant correlations (0.256–0.379, *p* ≤ 0.01) were found between the variables: age vs. years practicing CrossFit^®^ training; profession physical activity level vs. years practicing CrossFit^®^ training; profession physical activity level vs. CrossFit^®^ training level; profession physical activity level vs. annual CrossFit^®^ training competition; sport background vs. CrossFit^®^ training level; sport background vs. annual CrossFit^®^ training competition; CrossFit^®^ training level vs. weekly CrossFit^®^ training practice; CrossFit^®^ training level vs. CrossFit^®^ training practice duration; weekly CrossFit^®^ training practice vs. CrossFit^®^ training duration; weekly CrossFit^®^ training practice vs. annual CrossFit^®^ training competition; CrossFit^®^ training practice duration vs. annual CrossFit^®^ training competition; CrossFit^®^ training practice duration vs. performing warm-up; annual CrossFit^®^ training competition vs. cool-down strategies; performing warm-up vs. performing cool-down; and performing warm-up vs. cool-down strategies. Moreover, weak positive correlations (0.210–0.229) with low statistical significance (*p* ≤ 0.05) were found between the variables: age vs. performing cool-down; CrossFit^®^ training level vs. cool-down strategies; and performing cool-down vs. monitored by a health professional. Also, two weak negative correlations were found between sex and being monitored by a health professional (−0.219, *p* ≤ 0.05) and height and being monitored by a health professional (−0.225, *p* ≤ 0.05) variables. Negligible positive correlations (0.187–0.209, *p* ≤ 0.05) were found between the variables: sex vs. practicing other sports plus CrossFit^®^ training; profession physical activity level vs. performing warm-up; professional physical activity level vs. performing cool-down; years practicing CrossFit^®^ training vs. CrossFit^®^ training level; years practicing CrossFit^®^ training vs. CrossFit^®^ training practice duration; years practicing CrossFit^®^ training vs. annual CrossFit^®^ training competition; weekly CrossFit^®^ training practice vs. performing warm-up; cool-down strategies vs. being monitored by a health professional. Finally, a negligible negative correlation (−0.193, *p* ≤ 0.05) was found between sports background and presence and being monitored by a CrossFit^®^ training instructor (Table 6).

In the logistic regressions, two statistically significant models were identified in the variables related to injuries’ localization and type. Male CrossFit^®^ training practitioners were found to be 2.7 times more likely to suffer a tendon injury compared to their female counterparts (*p* = 0.012; R^2^ = 8%). Additionally, individuals with a sports background were 300% more susceptible to this injury type (*p* = 0.043; R^2^ = 5%). Conversely, those without a sports background were five times more likely to experience a muscular injury (*p* = 0.001; R^2^ = 13%). Concerning injury localizations, it was observed that having more years of CrossFit^®^ training practice led to a higher risk of shoulder injuries (1–3 years: OR = 3.014 and *p* = 0.125; 4–6 years: OR = 7.222 and *p* = 0.007; 7–≥10 years: OR = 4.333 and *p* = 0.049). Lastly, heavier training practitioners were 4.8 times more likely to suffer a knee injury compared to their lighter peers (*p* = 0.023; R^2^ = 10%). Table 7 provides a more detailed presentation of the described information.

## 4. Discussion

In this retrospective cross-sectional survey-based study, distinctive patterns emerged regarding injury types and localizations, shedding light on important sociodemographic characteristics that could influence the reporting of injuries. The prevalence of injuries among CrossFit^®^ practitioners was determined to be 39.9%, aligning with the existing literature where the median injury percentage was 35.6% (min: 12.8% [26]; max: 73.5% [47]) [10,11,12,15,20,23,24,25,26,28,40,47,51,52,53,54,55,56,57,58]. Although the exact injury incidence value was not calculable in this study, an estimated median injury incidence of 2.4/1000 h (min: 0.51 [10]; max: 18.9 [56]) was derived based on reported values in other studies [10,11,15,23,25,26,40,47,52,53,55,56,58]. Discrepancies in injury prevalence and incidence between studies may stem from study design differences (retrospective vs. prospective) and the applied injury definition [39,59].

Despite mainstream media scrutiny suggesting a high injury incidence in CrossFit^®^ training [10], evidence indicates that injury risk among CrossFit^®^ training participants is comparable to or lower than some common forms of exercises or sports [60,61]. For example, gymnastics [62] and other comparable weight-training sports [63] exhibit mean injury incidences of 2.2/1000 h and 2.9–7.5/1000 h, respectively. These findings emphasize that while injuries occur, they are often isolated incidents and should not be generalized to the broader CrossFit^®^ participant population [10]. In weight-training sports, musculotendinous injuries, particularly in the shoulder, lower back, knee, elbow, and/or wrist/hand, are common [63,64]. Similar trends were observed in CrossFit^®^, where the most prevalent injuries were localized in the shoulder, elbow, lower back, and knee. Notably, almost half of the injuries were in the shoulder (46.1%), consistent with findings in other studies within the same sports population [1,10,11,20,23,25,28,40,52,53,54,55,56,58,65]. Musculotendinous injuries, especially tendon-related (40.9%), dominated the injury types, attributable to the sport’s specific characteristics, including repetitive exercises, improper form, exercise types, previous injuries, training hours, and experience [22,39,66].

CrossFit^®^ training, characterized by its functional, varied, and high-intensity nature, involves rapid, repetitive movements with limited recovery time [20,21]. This demands the execution of advanced-level techniques at a fast pace, often with heavy loads, contributing to early fatigue, oxidative stress, inflammation, and unsafe movement execution. These factors collectively increase the risk of chronic/overuse injuries [13,20,37,54]. Complex coordinative movements combined with heavy external loads, particularly in more-experienced CrossFit^®^ practitioners, may explain the higher reporting of shoulder injuries in comparison to their less-experienced peers (4–6 years: OR = 7.222; 7–≥10 years: OR = 4.333). Additionally, the prevalence of shoulder injuries in Gymnastics (49.1%) and Weightlifting (37.8%) exercises aligns with literature findings attributing these movements as primary causes of shoulder injuries [65]. Improper form at overhead weight exercises can result in hyperflexion, internal rotation, and abduction of the shoulder, resulting in extreme forces on the joint and surrounding musculature [47]. This may lead to shoulder internal impingement (due to the pathologic contact between the glenoid and the humerus), which inevitably leads to injuries such as rotator cuff tears or tendinitis, labral tears, subacromial bursitis, and scarring of the capsule [66]. Furthermore, practitioners with shoulder pain show scapular girdle instability, especially due to reduced activation of the lower trapezius [67]. This is particularly worrying since the glenohumeral joint is already naturally unstable. It relies on the labrum, rotator cuff, and other surrounding musculature to keep the humeral head centered on the glenoid [66]. So, extraphysiologic exercises with a heavy load that uses momentum to carry the shoulder into abduction and external rotation (especially those that place the shoulder beyond the normal arc of motion) pose the highest risk for injuries such as luxations, fractures, rotator cuff tears, nerve palsies, bone loss, chronic instability, chondral wear, and eventually dislocation arthropathy [35,66]. The reduced body segment control may also explain why a significant number of shoulder injuries were found in weightlifting and gymnastics-related movements [68]. This is evidenced when experience is added, as it was found that rotator muscles in shoulder joints showed greater power and torque values in advanced competitive level CrossFit^®^ practitioners compared to those at a lower competitive level (i.e., beginner or intermediate) [69]. Moreover, the participants who competed at advanced levels exhibited a greater imbalance of peak torque between the muscles responsible for the external–internal diagonal and external–internal rotational movements of the shoulder [69]. This imbalance significantly increases the risk of developing a shoulder injury [69,70]. Therefore, it is recommended that novice practitioners limit their participation in high-intensity shoulder exercises that may place excessive stress on anatomical structures. For this population, scalable training is crucial [20,24,25,28]. Instructors should pay close attention to managing training volume, weight progression, and ensuring proper execution of movements/exercises, providing supervision and corrections as needed, and ultimately creating specific practitioners’ profile(s) and balanced training group(s) [71]. Moreover, conducting a preliminary assessment to evaluate practitioners’ readiness for CrossFit^®^ activities—such as physical condition and medical background—would be beneficial [71]. Additionally, given the complexity of these movements, prior proper preparation is essential. Implementing a program such as MobilityWOD could play a significant role in preventing these injuries [72]. This program includes self-myofascial release techniques, low-intensity resistance training (using resistance bands), and mobilization exercises [72]. All of these techniques may address specific needs, such as improving mobility, strengthening stabilizing muscles, and reducing the risk of shoulder injuries.

Lower lumbar spine injuries in CrossFit^®^ may be linked to specific exercises like squats, deadlifts, or tire flips, which entail repetitive flexo-extension and/or hyperextension with heavy loads and high speeds, demanding precise spinal alignment throughout each repetition [39]. The cumulative fatigue induced by a substantial number of repetitions can compromise proper technique, increasing the susceptibility to back injuries. The knee assumes a crucial role in load-carrying, being a particular load-bearing joint in which soft tissues function as the major stabilizing factors, especially the quadriceps. Excessive fatigue in this muscle group may adversely impact the lifting technique, leading to a stoop (back) lift rather than a proper squat lift, increasing the likelihood of knee and lower-back injuries [39]. In weight-training sports, high-bar or front squats may require greater knee extensor torques and produce greater mean compressive patellofemoral forces than low-bar squats [63,64]. On the other hand, low-bar squats result in a greater forward trunk inclination, that may require a higher trunk stabilization [63]. The execution of CrossFit^®^ exercises, including complex overhead movements, high-bar back squats, and rapid transitions, mirrors these patterns. It is highly recommended that a certified CrossFit^®^ instructor is always present in these (heavy) weight exercises, to monitor weight progression and ensure proper movement execution [34]. The instructor should provide continuous supervision, offer guidance, and make necessary corrections to promote safe and effective training [71]. Given the intensity and physical demands of these exercises, managing training volume is critical. These types of exercises should be performed sparingly, avoiding excessive frequency to reduce the risk of injury [10,40].

Among the studied variables, the most significant predictors of injury reporting were directly related to sports practice. Individuals with more experience (*p* = 0.006) and higher training frequency (*p* = 0.003) tended to report more injuries. For instance, practitioners with over 3 years of CrossFit^®^ experience or those training more than 11 h per week showed increased odds of injury [12]. A longer history of experience with CrossFit^®^ training was associated with higher injury odds [10,24,28]. This increased risk with greater exposure to physical activity aligns with the likelihood of previous injuries, making individuals more susceptible to reinjuries or the development of new ones [56,57]. Moreover, practitioners engaged in annual competitions at a higher level demonstrated a positive correlation with increased training volume and experience. This aligns with studies indicating that competitors have higher injury rates, longer CrossFit^®^ practice duration, and greater athlete training hours compared to non-competitors [15,25]. Participation in competitions, along with factors like rest per week, duration of training per session, and frequency of training per week, were statistically significant factors for injury [1]. Competitive-level athletes exhibited a five to seven times higher probability of injuries compared to beginners [11,12]. The heightened exposure to training intensity, loads, and complexity among competitive athletes may contribute to these findings [40,55].

While no statistically significant differences were found in the cross-level between practitioners reporting injuries and those who did not in this study, the proximity of the *p*-value (0.051) suggests that cross-level practice should be considered when evaluating injuries in this sport. Novice practitioners may face higher injury risks due to fewer training routines, fewer sport-specific skills, inadequate exercise chronic adaptations, and improper exercise execution [20,23,32,40,54,55,73,74]. This resonates with the proposed “training-injury prevention paradox”, emphasizing that injuries result not from higher training loads but from inappropriate progression plans causing excessive and rapid load increases, particularly in physically unprepared athletes [15,75]. Awareness of this issue among practitioners is evident, with reported causes including improper form, fatigue, and excessively heavy loads [20,65]. Therefore, to mitigate injuries, it is critical to have coaches/instructors specialized in evaluating practitioners individually, scalable training, and supervising the execution of the exercise (ensuring that they are performed with better quality, technique, and safety) [13,15,35,55,56]. The reported presence and monitoring by an instructor (88.5%) may have contributed to the relatively low injury reporting in the novice practitioners’ sample. Additionally, the implementation of an injury prevention program, particularly for shoulder, lower back, and knee, focusing on musculotendinous disorders, is recommended.

Certain personal characteristics, particularly sex and weight, emerged as potential risk factors. Males and heavier individuals were found to have a higher risk of suffering tendon and knee injuries (OR: 2.7, *p* = 0.012; and OR: 4.8, *p* = 0.023, respectively). Similar trends were reported in other studies [23,25,55]. The association between male sex, higher body weight, and increased risk of injury may be attributed to larger size individuals training with greater loads [39]. However, the limited literature on these personal characteristics necessitates further investigation to establish a definitive understanding of their impact on injury risk. Based on the study’s findings, additional research is recommended, particularly a more detailed investigation of the personal and sport-related factors identified, a deeper exploration of the relationship between CrossFit^®^ movements and the incidence of various injury types, the use of advanced technology for biomechanical analysis of the associated movements for a better understanding of these injury patterns and causes, and the development of injury prevention plans tailored specifically to the needs of CrossFit^®^ practitioners.

### Limitations

This study has limitations that may influence the interpretation and generalization of its findings. Firstly, the small number of valid participants restricts the generalizability of the findings. A larger sample, with different participants and personal experiences, could influence the health/injury patterns. Secondly, the construction and operationalization of the questionnaire present significant drawbacks: (a) it lacked sensitivity to capture all injuries, as the closed-answer options did not allow for specific details when selecting “other”; (b) due to the choice of question-and-answer format, which only included closed-ended responses, in-depth information (qualitative data) could not be obtained; (c) due to constraints in the questionnaire software, respondents could only explore one injury, limiting a comprehensive understanding of all the injuries experienced by CrossFit^®^ practitioners over the years; (d) due to the absence of a defined time range, the incidence of injuries could not be determined, limiting the understanding of the most significant injuries within the sample; (e) the questionnaire could be made more sensitive to identify the specific exercises and biomechanics associated with particular injury types and locations (e.g., squats, deadlifts, snatches, clean and jerks, pull-ups, ring muscle-ups, rope climbs, rope jumping, kettlebell swings, box jumps, and others); (f) it may also be important to explore other variables related to injuries and their patterns, such as recovery programs, nutrition, prevention strategies, and psychological training. The third and final limitation arises from the retrospective and self-reporting nature of the questionnaire, potentially introducing reporting errors due to participants’ recall limitations and varying levels of health literacy and knowledge.

## 5. Conclusions

In conclusion, up to 40% of CrossFit^®^ training practitioners may experience one to three sports-related injuries. The most prevalent injuries involve the shoulder and musculotendinous regions. Reporting of sports-related injuries appears to be influenced by variables such as years of practice, weekly training frequency, gender, sports background, and weight. Coaches and practitioners should prioritize exercise progression and execution, especially in overhead complex exercises.

## Figures and Tables

**Table 1:** Exercises/drills included in the warm-up sessions.

Static Stretching	Dynamic Stretching	Sport-Specific	Core	Running	Mobility	Running Drills	Strengthening	Balance	Self-Massage	n (%)
					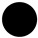					14 (6.1)
	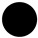				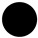					13 (5.7)
	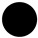	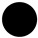			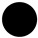					11 (4.8)
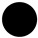	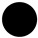				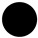					9 (4.0)
		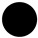			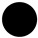					8 (3.5)
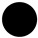	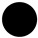	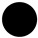	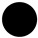		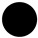					7 (3.1)
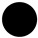	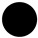	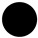			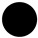					7 (3.1)
	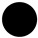	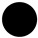	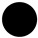		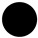		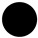			6 (2.6)
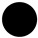					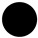					5 (2.2)
			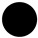		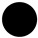					5 (2.2)
	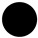									4 (1.8)
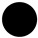	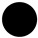									4 (1.8)
					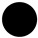	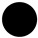				4 (1.8)
	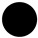	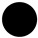	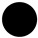		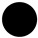					4 (1.8)
		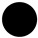								4 (1.8)
				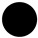	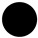					3 (1.3)
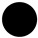	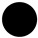			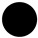	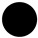					3 (1.3)
	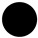		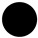		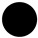					3 (1.3)
	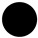				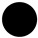		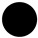			3 (1.3)
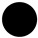		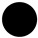			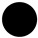					3 (1.3)
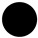	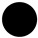	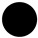								2 (0.9)
	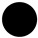	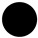	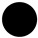							2 (0.9)
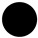	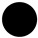				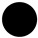	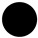				2 (0.9)
	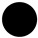		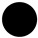		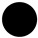	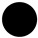				2 (0.9)
							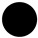			2 (0.9)
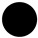	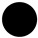		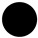		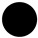					2 (0.9)
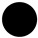	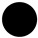	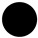	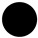		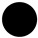		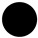			2 (0.9)
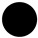	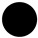	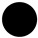	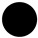		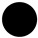		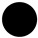	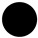		2 (0.9)
	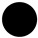	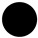	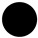		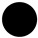		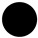		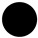	2 (0.9)
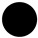			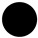		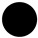					2 (0.9)
			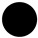		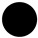		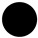			2 (0.9)
		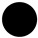	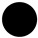		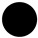		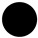			2 (0.9)
		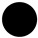			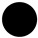		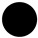			2 (0.9)

Note: Of the 114 different warm-up variations, 81 (71.1%) were chosen once.

**Table 2:** CrossFit^®^ training practitioner’s sport and sociodemographic characteristics and risk factors.

Variable	Total (n = 288 (100%)) n(%)	Unreported Injury (n = 173 (60.1%)) n(%)	Reported Injury (n = 115 (39.9%)) n(%)	*p* Injury vs. No Injury
Age group (years)				0.727 *
*18–24*	15 (5.2)	10 (5.8)	5 (4.3)	
*25–29*	52 (18.1)	30 (17.3)	22 (19.1)	
*30–34*	55 (19.1)	45 (26)	24 (20.9)	
*35–39*	69 (24.0)	36 (20.8)	26 (22.6)	
*40–44*	26 (9.0)	15 (8.7)	11 (9.6)	
*45–50*	9 (3.1)	7 (4.0)	2 (1.7)	
*+50*				
Sex				0.614 **
*Female*	140 (48.6)	82 (47.4)	58 (50.4)	
*Male*	148 (51.4)	91 (52.6)	57 (49.6)	
Body weight (kilograms)				0.612 *
*−50*	6 (2.1)	4 (2.3)	2 (1.7)	
*50–74*	158 (54.9)	97 (56.1)	61 (53.0)	
*75–100*	114 (39.6)	65 (37.6)	49 (42.6)	
*+100*	10 (3.5)	7 (4.0)	3 (2.6)	
Body height (meters)				0.618 *
*−1.50*	2 (0.7)	2 (1.2)	0 (0.0)	
*1.50–1.74*	180 (62.5)	109 (63)	71 (61.7)	
*1.75–2.00*	105 (36.5)	61 (35.3)	44 (38.3)	
*+2.00*	1 (0.3)	1 (0.6)	0 (0.0)	
Profession physical activity level				0.505 *
*Sedentary*	64 (22.2)	35 (20.2)	29 (25.2)	
*Sitting and walking, without physical efforts*	113 (39.2)	70 (40.5)	43 (37.4)	
*Sitting and walking, with moderate physical efforts*	32 (11.1)	17 (9.8)	15 (13)	
*Sitting and walking, with heavy physical efforts*	5 (1.7)	4 (2.3)	1 (0.9)	
*Standing and walking, without physical efforts*	18 (6.3)	13 (7.5)	5 (4.3)	
*Standing and walking, with moderate physical efforts*	39 (13.5)	26 (15.0)	13 (11.3)	
*Standing and walking, with heavy physical efforts*	17 (5.9)	8 (4.6)	9 (7.8)	
Sport or physical activity background				0.199 **
*Yes*	219 (76.0)	127 (73.4)	92 (80.0)	
*No*	69 (24.0)	46 (26.6)	23 (20.0)	
Years practicing CrossFit^®^ training				0.006 *
*−1*	65 (22.6)	49 (28.3)	16 (13.9)	
*1–3*	94 (32.6)	55 (31.8)	39 (33.9)	
*4–6*	73 (25.3)	41 (23.7)	32 (27.8)	
*7–10*	48 (16.7)	23 (13.3)	25 (21.7)	
*+10*	8 (2.8)	5 (2.9)	3 (2.6)	
CrossFit^®^ training competitive level				0.051 *
*Non-competitive*	197 (68.4)	125 (72.3)	72 (62.6)	
*Competitive, recreational*	45 (15.6)	26 (15)	19 (16.5)	
*Competitive, beginner*	25 (8.7)	14 (8.1)	11 (9.6)	
*Competitive, advanced*	21 (7.3)	8 (4.6)	13 (11.3)	
CrossFit^®^ training weekly practice				0.003 *
*Once per week*	8 (2.8)	8 (4.6)	0 (0.0)	
*2–3 times per week*	90 (31.3)	62 (35.8)	28 (24.3)	
*4–6 times per week*	180 (62.5)	98 (56.6)	82 (71.3)	
*7 or more times a week*	10 (3.5)	5 (2.9)	5 (4.3)	
CrossFit^®^ training practice duration (minutes)				0.615 *
*30–59*	178 (61.8)	104 (60.1)	74 (64.3)	
*60–89*	100 (34.7)	65 (37.6)	35 (30.4)	
*90–120*	10 (3.5)	4 (2.3)	6 (5.2)	
Annual CrossFit^®^ training competitions participations				0.076 *
*Do not compete*	197 (68.4)	125 (72.3)	72 (62.6)	
*1*	27 (9.4)	15 (8.7)	12 (10.4)	
*2*	37 (12.8)	20 (11.6)	17 (14.8)	
*3*	17 (5.9)	7 (4.0)	10 (8.7)	
*4*	7 (2.4)	5 (2.9)	2 (1.7)	
*5*	1 (0.3)	0 (0.0)	1 (0.9)	
*+5*	2 (0.7)	1 (0.6)	1 (0.9)	
Warm-up before training/competitions?				0.808 *
*No*	56 (19.4)	33 (19.1)	23 (20.0)	
*Yes,* −*10 min*	140 (48.6)	83 (48.0)	57 (49.6)	
*Yes, 10–20 min*	85 (29.5)	55 (31.8)	30 (26.1)	
*Yes, 21–30 min*	6 (2.1)	2 (1.2)	4 (3.5)	
*Yes, +30 min*	1 (0.3)	0 (0.0)	1 (0.9)	
Warm-up exercises used				
*Running*	32 (3.9)	20 (4.1)	12 (3.5)	0.766 **
*Sprint*	8 (1.0)	6 (1.2)	2 (0.6)	0.382 **
*Running drills*	56 (6.8)	34 (7.0)	22 (6.4)	0.913 **
*Mobility exercises*	198 (23.9)	117 (24.2)	81 (23.5)	0.615 **
*Dynamic stretching*	152 (18.4)	86 (17.8)	66 (19.1)	0.201 **
*Static stretching*	90 (10.9)	51 (10.6)	39 (11.3)	0.427 **
*Strengthening exercises*	47 (5.7)	26 (5.4)	21 (6.1)	0.467 **
*Core exercises*	90 (10.9)	50 (10.4)	40 (11.6)	0.292 **
*Balance exercises*	28 (3.4)	15 (3.1)	13 (3.8)	0.460 **
*Sport-specific exercises*	102 (12.3)	60 (12.4)	42 (12.2)	0.749 **
*Plyometrics*	11 (1.3)	9 (1.9)	2 (0.6)	0.133 **
*Self-massage*	8 (1.0)	4 (0.8)	4 (1.2)	0.555 **
*Other*	6 (0.7)	5 (1.0)	1 (0.3)	0.240 **
Cool-down after training/competitions?				0.456 *
*No*	99 (34.4)	57 (32.9)	42 (36.5)	
*Yes, −10 min*	163 (56.6)	99 (57.2)	64 (55.7)	
*Yes, 10–20 min*	23 (8.0)	15 (8.7)	8 (7.0)	
*Yes, 21–30 min*	1 (0.3)	0 (0.0)	1 (0.9)	
*Yes, +30 min*	2 (0.7)	2 (1.2)	0 (0.0)	
Cool-down strategies used				0.305 **
*Stretching*	122 (64.6)	80 (69.0)	42 (57.5)	
*Electric stimulation*	2 (1.1)	0 (0.0)	2 (2.7)	
*Manual massage*	5 (2.6)	3 (2.6)	2 (2.7)	
*Massage guns*	8 (4.2)	4 (3.4)	4 (5.5)	
*Active recovery*	25 (13.2)	13 (11.2)	12 (16.4)	
*Passive recovery/Rest*	6 (3.2)	4 (3.4)	2 (2.7)	
*Foam roller*	11 (5.8)	4 (3.4)	7 (9.6)	
*Nutrition/Supplementation*	8 (4.2)	6 (5.2)	2 (2.7)	
*Other*	2 (1.1)	2 (1.7)	0 (0.0)	
Presence and being monitored by a CrossFit^®^ training instructor				0.226 **
*No*	7 (2.4)	2 (1.2)	5 (4.3)	
*Yes, and non-monitored*	26 (9.0)	16 (9.2)	10 (8.7)	
*Yes, and monitored*	255 (88.5)	155 (89.6)	100 (87.0)	
Monitored by a health professional				0.903 **
*No*	154 (53.5)	92 (53.2)	62 (53.9)	
*Yes*	134 (46.5)	81 (46.8)	53 (46.1)	
Practice other sports or physical activity?				0.891 *
*No*	170 (59.0)	102 (59.0)	68 (59.1)	
*Yes, once per week*	68 (23.6)	40 (23.1)	28 (24.3)	
*Yes, 2 times per week*	27 (9.4)	16 (9.2)	11 (9.6)	
*Yes, 3 times per week*	10 (3.5)	7 (4.0)	3 (2.6)	
*Yes, more than 3 times per week*	13 (4.5)	8 (4.6)	5 (4.3)	

Note: Bold—Significant statistic differences; * Mann–Whitney U test; ** Chi-square test.

**Table 3:** CrossFit^®^ training practitioner’s reported injuries characterization (n = 115).

Variable	n (%)
N° of injuries	
*1*	56 (48.7)
*2*	40 (34.8)
*3*	12 (10.4)
*4*	3 (2.6)
*5*	0 (0.0)
*+5*	4 (3.5)
Injury localization	
*Head*	2 (1.7)
*Cervical Spine*	2 (1.7)
*Upper Back*	1 (0.9)
*Thoracic Spine*	1 (0.9)
*Middle Back*	2 (1.7)
*Chest/Ribs*	2 (1.7)
*Lower Back*	10 (8.7)
*Lumbar Spine*	3 (2.6)
*Shoulder*	53 (46.1)
*Elbow*	9 (7.8)
*Forearm (posterior)*	1 (0.9)
*Wrist*	2 (1.7)
*Hand/Fingers*	1 (0.9)
*Pelvis/Groin*	1 (0.9)
*Thigh (anterior)*	2 (1.7)
*Thigh (posterior)*	1 (1.7)
*Knee*	13 (11.3)
*Leg (anterior)*	2 (1.7)
*Leg (posterior)*	1 (0.9)
*Ankle*	1 (0.9)
*Foot/Fingers*	4 (3.5)
*Other*	1 (0.9)
Injury type	
*Bursitis*	3 (2.6)
*Concussion*	1 (0.9)
*Organ Trauma*	1 (0.9)
*Laceration/Abrasion/Bleeding*	3 (2.6)
*Joint Injury*	9 (7.8)
*Fascial Injury*	4 (3.5)
*Ligamentous Injury*	5 (4.3)
*Meniscal Injury*	2 (1.7)
*Muscular Injury*	32 (27.8)
*Bone Injury*	4 (3.5)
*Tendon Injury*	47 (40.9)
*Pain*	1 (0.9)
*Other*	3 (2.6)
Injury occurrence situation	
*Warm-up (training)*	5 (4.3)
*Cool-down (training)*	6 (5.2)
*During training*	96 (83.5)
*During competition*	8 (7.0)
Injury CrossFit^®^ Training exercise	
*Endurance*	4 (3.5)
*Gymnastics*	35 (30.4)
*Olympic Lifting*	24 (20.9)
*Power Lifting*	32 (27.8)
*Other*	20 (17.4)
Return-to-sport duration	
*−1 week*	28 (24.3)
*1–2 weeks*	22 (19.1)
*3–4 weeks*	21 (18.3)
*1–3 months*	25 (21.7)
*4–6 months*	10 (8.7)
*7–12 months*	7 (6.1)
*+1 year*	2 (1.7)
Injury history	
*Recurrence*	23 (20.0)
*First time*	92 (80.0)
Injury management	
*No intervention*	5 (4.3)
*Active self-management*	11 (9.6)
*Self-Medication/Supplementation*	4 (3.5)
*Physiotherapist*	52 (45.2)
*Physician (surgery)*	5 (4.3)
*Physician (injection)*	1 (0.9)
*Physician (medication)*	15 (13.0)
*Non-conventional medicine*	10 (8.7)
*Rest*	12 (10.4)

**Table 4:** Injury type and localization distribution (n = 115).

Injury Type; Localization	n (%)
*Bursitis*	
Shoulder	3 (100)
*Concussion*	
Head	1 (100)
*Organ Trauma*	
Other	1 (100)
*Laceration/Abrasion/Bleeding*	
Head	1 (33.3)
Shoulder	1 (33.3)
Leg (posterior)	1 (33.3)
*Joint*	
Cervical Spine	1 (11.1)
Lumbar Spine	1 (11.1)
Shoulder	3 (33.3)
Elbow	3 (33.3)
Wrist	1 (11.1)
*Fascial*	
Shoulder	1 (25)
Knee	1 (25)
Foot/Fingers	2 (50)
*Ligamentous*	
Shoulder	2 (40)
Hand/Fingers	1 (20)
Knee	2 (40)
*Meniscal*	
Knee	2 (100)
*Muscular*	
Upper Back	1 (3.1)
Middle Back	2 (6.3)
Chest/Ribs	1 (3.1)
Lower Back	9 (28.1)
Shoulder	13 (40.6)
Forearm (posterior)	1 (3.1)
Thigh (anterior)	1 (3.1)
Thigh (posterior)	1 (3.1)
Leg (anterior)	2 (6.3)
Foot/Fingers	1 (3.1)
*Bone*	
Chest/Ribs	1 (20)
Lumbar Spine	1 (20)
Thigh (anterior)	1 (20)
Knee	1 (20)
*Tendon*	
Lower Back	1 (2.1)
Shoulder	29 (61.7)
Elbow	6 (12.8)
Wrist	1 (2.1)
Pelvis/Groin	1 (2.1)
Knee	7 (14.9)
Ankle	1 (2.1)
Foot/Fingers	1 (2.1)
*Pain*	
Cervical Spine	1 (100)
*Other*	
Thoracic Spine	1 (33.3)
Lumbar Spine	1 (33.3)
Shoulder	1 (33.3)

**Table 5:** Injury type and localization distribution by situation, exercise, return-to-sport, clinal history, and management (n = 115).

Injury Localization	Situation				Exercise					Return-to-Sport							Clinical History		Management								
	*Warm-Up (Training)*	*Cool-Down (Training)*	*During Training*	*During Competition*	*Endurance*	*Gymnastic*	*Olympic Lifting*	*Power Lifting*	*Other*	*−1 Week*	*1–2 Weeks*	*3–4 Weeks*	*1–3 Months*	*4–6 Months*	*7–12 Months*	*+ 1 Year*	*Recurrence*	*First Time*	*No Intervention*	*Self-Management*	*Self-Medication*	*Physio* *therapist*	*Physician (Surgery)*	***Physician (Injection)***	***Physician (Medication)***	***Non-Conventional Medicine***	***Rest***
*Head*	-	-	2	-	-	1	-	1	-	-	-	1	1	-	-	-	-	2	-	-	-	-	-	-	1	-	1
*Cervical Spine*	-	-	2	-	-	-	1	-	1	-	-	1	1	-	-	-	-	2	-	-	-	1	-	-	-	-	1
*Upper Back*	-	-	1	-	-	-	-	1	-	-	1	-	-	-	-	-	1	-	-	-	-	1	-	-	-	-	-
*Thoracic Spine*	-	-	1	-	-	-	-	1	-	-	-	-	1	-	-	-	-	1	-	-	-	1	-	-	-	-	-
*Middle Back*	-	1	1	-	-	1	-	1	-	1	1	-	-	-	-	-	1	1	-	-	1	-	-	-	-	1	-
*Chest/Ribs*	-	-	2	-	-	2	-	-	-	-	1	-	1	-	-	-	-	2	-	-	1	1	-	-	-	-	-
*Lower Back*	-	-	10	-	-	-	2	8	-	3	4	2	1	-	-	-	1	9	-	4	-	1	-	-	3	-	2
*Lumbar Spine*	1	-	2	-	-	-	2	-	1	-	-	1	1	-	1	-	-	3	-	-	-	1	-	-	2	-	-
*Shoulder*	2	1	43	7	-	26	11	9	7	16	9	12	6	6	3	1	17	36	3	3	2	25	3	1	4	8	4
*Elbow*	-	-	8	1	-	1	6	1	1	1	2	2	3	1	-	-	1	8	-	2	-	6	-	-	1	-	-
*Forearm (posterior)*	-	1	-	-	-	-	-	1	-	1	-	-	-	-	-	-	-	1	-	-	-	1	-	-	-	-	-
*Wrist*	-	-	2	-	-	1	-	1	-	-	-	-	2	-	-	-	-	2	-	-	-	2	-	-	-	-	-
*Hand/Fingers*	-	-	1	-	-	-	-	-	1	-	-	-	1	-	-	-	-	1	-	-	-	-	-	-	-	-	1
*Pelvis/Groin*	-	-	1	-	-	-	-	1	-	-	-	-	-	-	1	-	-	1	-	-	-	1	-	-	-	-	-
*Thigh (anterior)*	-	-	2	-	1	1	-	-	-	-	-	-	1	-	1	-	-	2	-	-	-	1	-	-	-	-	1
*Thigh (posterior)*	-	-	1	-	1	-	-	-	-	-	-	-	1	-	-	-	-	1	-	-	-	1	-	-	-	-	-
*Knee*	2	-	11	-	2	-	2	5	4	2	3	1	4	1	1	1	1	12	-	1	-	6	2	-	2	-	2
*Lower Leg (anterior)*	-	-	2	-	-	-	-	1	1	1	-	-	1	-	-	-	1	1	2	-	-	-	-	-	-	-	-
*Lower Leg (posterior)*	-	1	-	-	-	-	-	1	-	1	-	-	-	-	-	-	-	1	-	-	-	1	-	-	-	-	-
*Ankle*	-	-	1	-	-	-	-	-	1	1	-	-	-	-	-	-	-	1	-	1	-	-	-	-	-	-	-
*Foot/Fingers*	-	2	2	-	-	2	-	-	2	1	1	-	-	2	-	-	-	4	-	-	-	2	-	-	1	1	-
*Other*	-	-	1	-	-	-	-	-	1	-	-	1	-	-	-	-	-	1	-	-	-	-	-	-	1	-	-
Injury Type																											
*Bursitis*	1	-	2	-	-	2	-	1	-	1	-	-	-	1	-	1	-	3	-	-	-	3	-	-	-	-	-
*Concussion*	-	-	1	-	-	-	-	1	-	-	-	1	-	-	-	-	-	1	-	-	-	-	-	-	1	-	-
*Organ*	-	-	1	-	-	-	-	-	1	-	-	1	-	-	-	-	-	1	-	-	-	-	-	-	1	-	-
*Laceration*	-	1	2	-	-	1	-	1	1	2	-	-	1	-	-	-	-	3	1	-	-	1	-	-	-	-	1
*Joint*	1	-	6	2	-	1	3	2	3	1	2	2	3	1	-	-	-	9	-	1	-	3	-	1	2	1	1
*Fascial*	-	1	3	-	-	1	-	2	1	2	1	-	1	-	-	-	-	4	-	-	-	1	1	-	2	-	-
*Ligamentous*	2	-	2	1	1	2	-	-	2	-	1	-	3	-	1	-	-	5	-	1	-	2	1	-	-	-	1
*Meniscal*	-	-	2	-	1	-	-	1	-	-	-	-	2	-	-	-	-	2	-	-	-	-	2	-	-	-	-
*Muscular*	-	3	28	1	1	9	5	15	2	12	9	2	5	4	-	-	11	21	3	3	1	15	-	-	3	4	3
*Bone*	-	-	4	-	1	1	1	1	-	-	-	1	2	-	1	-	-	4	-	-	1	1	-	-	1	-	1
*Tendon*	1	-	42	4	-	18	14	7	8	10	9	12	7	4	4	1	11	36	1	6	2	23	1	-	5	5	4
*Pain*	-	-	1	-	-	-	-	-	1	-	-	1	-	-	-	-	-	1	-	-	-	1	-	-	-	-	-
*Other*	-	1	2	-	-	-	1	1	1	-	-	1	1	-	1	-	1	2	-	-	-	2	-	-	-	-	1

**Table 6:** Spearman correlations between the sociodemographic, sport, and injury variables (n = 115).

Variables	Age	Sex	Body Weight	Body Height	Professional Physical Activity Level	Sport Background	Years Practicing Cross	Cross Level	Weekly Cross Practice	Cross Practice Duration	Annual Cross Competitions	Perform Warm-Up	Perform Cool-Down	**Cool-Down Strategies**	**Presence and Monitored by an Instructor**	**N° Injuries Cross-Related**	**Injury Localization**	**Injury Cross Activity**
Body weight	-	0.558 **	-	-	-	-	-	-	-	-	-	-	-	-	-	-	-	-
Body height	-	0.723 **	0.605 **	-	-	-	-	-	-	-	-	-	-	-	-	-	-	-
Years practicing CrossFit^®^	0.332 **	-	-	-	0.256 **	-	-	-	-	-	-	-	-	-	-	-	-	-
CrossFit^®^ Level	-	-	-	-	0.379 **	0.274 **	0.194 *	-	-	-	-	-	-	-	-	-	-	-
Weekly CrossFit^®^ practice	-	-	-	-	-	-	-	0.348 **	-	-	-	-	-	-	-	-	-	-
CrossFit^®^ practice duration	-	-	-	-	-	-	0.196 *	0.284 **	0.257 **	-	-	-	-	-	-	-	-	-
Annual CrossFit^®^ competitions	-	-	-	-	0.365 **	0.289 **	0.207 *	0.955 **	0.314 **	0.295 **	-	-	-	-	-	-	-	-
Perform warm-up	-	-	-	-	0.205 *	-	-	-	0.187 *	0.333 **	-	-	-	-	-	-	-	-
Perform cool-down	0.227 *	-	-	-	0.198 *	-	-	-	-	-	-	0.368 **	-	-	-	-	-	-
Cool-down strategies	-	-	-	-	-	-	-	0.229 *	-	-	0.260 **	0.269 **	0.857 **	-	-	-	-	-
Presence and monitored by an instructor	-	-	-	-	-	−0.193 *	-	-	-	-	-	-	-	-	-	-	-	-
Monitored by a health professional	-	−0.219 *	-	−0.225 *	-	-	-	-	-	-	-	-	0.210 *	0.209 *	-	-	-	-
Practice other sports plus CrossFit^®^	-	0.191 *	-	-	-	-	-	-	-	-	-	-	-	-	-	-	-	-
N° injuries CrossFit^®^-related	0.202 *	-	0.221 *	-	0.274 **	-	0.454 **	-	-	-	-	-	-	-	−0.190 *	-	-	-
Injury type	-	-	-	-	-	-	-	-	-	-	-	-	-	-	-	-	-	-
Injury occurrence situation	-	-	-	-	0.186 *	-	-	0.323 **	-	-	0.337 **	-	-	-	-	-	−0.208 *	-
Injury CrossFit^®^ activity	-	-	-	-	−0.249 **	-	−0.246 **	-	-	-	-	-	-	-	-	−0.203 *	0.248 **	-
Return-to-sport duration	-	-	-	-	-	-	0.190 *	-	-	-	-	-	-	-	-	-	-	−0.191 *

Note: Only the significant statistical correlations are displayed; ** *p* ≤ 0.01; * *p* ≤ 0.05.

**Table 7:** Regression models between the sociodemographic, sport, and injury variables (n = 115).

Injury (Present)	Factor—Level	Odds Ratio (95% CI)	*p*	*R* ^2 a^
Tendon				
	*Sex*		*0.012*	*0.075*
	Male	2.680 [1.243; 5.777]		
	Female	Reference		
	*Sport Background*		*0.043*	*0.053*
	Yes	3.024 [1.035; 8.837]		
	No	Reference		
Muscular				
	*Sport Background*		*0.001*	*0.129*
	No	4.995 [1.900; 13.132]		
	Yes	Reference		
Shoulder				
	*Years Practicing Cross*		*0.045*	*0.104*
	1–3	3.014 [0.737; 12.325]	0.125	
	4–6	7.222 [1.703; 30.637]	0.007	
	7–≥10	4.333 [1.009; 18.615]	0.049	
	−1	Reference		
Knee				
	*Body Weight (Kg)*		*0.023*	*0.102*
	75–≥100	4.762 [1.236; 18.353]		
	≤50–74	Reference		

Note: ^a^ Nagelkerke R^2^. Only the significant statistical models are displayed.

## Data Availability

The data presented in this study are available on request from the corresponding author.
